# Relational skill training for patient engagement and the creation of a trauma-informed critical care

**DOI:** 10.1177/08404704231215461

**Published:** 2023-11-18

**Authors:** Laura Istanboulian, Tasneem Master, Christine Devine, Lorrie Hamilton

**Affiliations:** 1 7984 Toronto Metropolitan University, Toronto, Ontario, Canada.; 2 27374 Michael Garron Hospital, Toronto, Ontario, Canada.

## Abstract

Patients and families in critical care have a high likelihood of previous and re-experienced trauma. Unaddressed, physical, and psychological impacts of these traumas can worsen outcomes for patients and families. A trauma-informed care approach has been proposed for critical care; however, training programs do not include relational competencies or de-escalation techniques, risking the re-traumatization of patients and families in critical care and negatively impacting clinicians. This article describes a strategy that can be adopted by critical care teams towards the creation of a trauma-informed critical care unit including the use of a framework for relational training. Principles of relationship management and de-escalation are discussed with the use of a fictional exemplar scenario. Key messages include a call to action for relational training for care teams to enhance skilled relational engagement of patients and families. This article also highlights the foundational importance of policies supporting a trauma-informed approach in critical care.

## Introduction

Although Intensive Care Unit (ICU) clinicians are called to promote patient physical and psychological safety, their training does not include de-escalation and relational management techniques, perpetuating an untrained workforce and teams with inconsistent practices. To bridge this gap, critical care leadership can support unit- and/or institution-wide training of staff in de-escalation and relational skills. Training in relational skills for ICU teams can in this sense be an adjunct to trauma-informed care training and the creation of a trauma-informed unit.

Patients and families in the ICU have a high likelihood of previous or re-experienced trauma. Traumatic events may sometimes be the direct cause of ICU admission, such as when a patient is admitted after an assault or may be more distant in a patient’s history.^1^ Psychological trauma impacts two-thirds of Americans, suggesting that many ICU patients have a history of trauma.^1^ The DSM-5 definition of trauma requires “actual or threatened death, serious injury, or sexual violence.”^2^ Therefore, critical illness itself for many can be considered a traumatic event for patients and their families.^3,4^

Trauma can have lifelong physical and mental health effects on a person and their families.^5,6^ Conditions in the ICU such as lack of privacy, control, isolation, loud and disruptive sounds, inconsistent sleep, and delirium can be perceived as threatening to persons with a traumatic history and may trigger adaptive responses that manifest as behavioural, cognitive, social, and physical effects. These effects include agitation, defensiveness, anger, or withdrawal when interacting with the ICU team, which may complicate care and lead to conflict, but might also lead to alterations in care pathways.^1,3,7^ Relational skill building will not “solve” ICU conditions; however, it can help to improve relations between staff, patients, and family to prevent further re-traumatization that can be exacerbated by such conditions.

A trauma-informed care approach has been suggested for ICU. This approach recognizes the prevalence and impact of trauma on patients and families and aims to provide care without re-traumatization.^1^ Principles of trauma-informed care include safety; trustworthiness and transparency; peer support; collaboration and mutuality; empowerment, voice, and choice; and recognition of cultural, historical, and gender issues.^1^ Using a trauma-informed care approach in ICU can support physical recovery of patients and reduce risk of indirect harm due to agitation (e.g., risk of removing life-support equipment).^1^ Trauma-informed care also aligns with dominant ICU care paradigms such as patient and family-centred care.^8^ It also supports the minimization of physical and chemical restraint through the provision of relational and contextual alternative strategies.^9^

To address escalating violence in the workplace, multipronged programs are recommended that include workplace violence training for staff such as the Crisis Intervention Training Program.^10,11^ In addition to other guided lessons and training activities, this program includes discussions about how to apply de-escalation techniques in healthcare scenarios. The program describes the application of seven principles of relational management to prevent and address responsive behaviours and conflict with patients and families.^11^ These are power and equity; social exchange and reciprocity; empathy, caring, and acceptance; genuineness and openness; reading and responding to emotions; avoiding coercion; and setting limits and interpreting boundaries (Table 1). Not intended to be used out of the context of the full crisis intervention training or as an enumerated checklist, working through these principles together can help critical care teams understand patient and family responsive behaviours, prevent (re-) traumatization, improve relationships, and therefore support the foundation of trauma-informed care training and concepts in the ICU.

**Table 1.** Description of the seven principles of relational skills in crisis intervention training.^11^.

Principle	Description
Power and equity	Assess if the patient has sufficient self-determination in daily routines. Provide greater choices.
Social exchange and reciprocity	Assess if the relationship is imbalanced with insufficient positives and excessive negatives and create relationship balance by giving more positives and reducing unnecessary negatives.
Empathy, caring, and acceptance	Communicate empathy. Show caring by responding to needs/wants. Show acceptance by respecting rights. Use “active listening” skills.
Genuineness and openness	Assess feelings towards the patient and disclose feelings, personal thoughts, and information while maintaining appropriate professional boundaries.
Reading and responding to emotions	Assess emotions using target indicators and work through emotions with active listening. Work through initial emotional expression and then facilitate problem-solving by defining the problem, brainstorming, weighing alternatives, and planning implementation.
Avoiding coercion	Identify and don’t give into coercive behaviour. Avoid confrontation and redirect by actively listening and problem-solving.
Setting limits and interpreting boundaries	Assess inconsistencies between staff and between various situations. Set limits, behavioural expectations, and rules. Discuss specific rules and limits with other staff, write them down to ensure consistency, and review follow-through.

### Aim

Using a fictional exemplar scenario, we aimed to illustrate how crisis intervention training relational management principles can be applied to support team-based de-escalation, build relationships, prevent (re-)traumatization, and to contribute to the creation of a trauma-informed ICU.

## Methods

The author team includes three nurse leaders with ICU experience and one wellness specialist. Two members of the team have extensive experience providing crisis intervention training in the hospital setting using the Crisis Intervention Training Program Health Care Manual.^11^ This framework has been used at the site for several years as part of the workplace violence prevention program. Together the team created a completely fictional ICU exemplar case, discussed the case using the seven principles of relational skills of crisis intervention training, and identified specific individual and team strategies and techniques that could be used to address the identified issues, promote care within the exemplar, and contribute to a trauma-informed ICU.

### Exemplar scenario

A patient we have named Joy (she/her) in her 30s is admitted to ICU with acute respiratory failure after infection with COVID-19. Joy has a prolonged ICU admission due to multiple infections, severe deconditioning, and a sacral ulcer. Because Joy requires mechanical ventilation, she can’t vocalize and uses hand gestures and mouthing to communicate.

Joy’s mother we have named Agnes (she/her) visits daily and informs the team that the patient has a history of severe anxiety and depression. The team reports that she does not “participate in daily care” or physiotherapy sessions due to fatigue and pain despite encouragement. The staff frequently express frustration about working with Joy and Agnes, her mother. Agnes is frustrated with her lack of progress and has made several complaints to the unit manager about various aspects of her care.

Today, the physiotherapist enters the room and asks permission to get Joy into a chair. Joy nods her head to say “no.” Agnes says, “yes she will—get her in the chair.” The staff reply that they need the Joy’s permission, to which Agnes throws up her hands and yells “I have had just about enough of this, I want to speak to the manager, you are ignoring my daughter’s needs and making her get worse.” In response to Agnes’s physical and verbal expression, the staff leave the Joy’s room.

## Results

This fictional scenario describes a conflict in ICU between the patient Joy, the patient’s mother Agnes, and healthcare team. There is a history of disagreement and complaint preceding this interaction, and then Agnes starts to show agitated behaviours, suggesting this situation has the potential to escalate. Below, we present this author team’s summary of de-escalate strategies and ways to improve the patient-family-clinician relationship. We feel it is important to note to the reader that we acknowledge that other teams may have additional or different recommendations based on their interpretation of the scenario, knowledge, and experience.

### Power and equity

The team discussed the importance of providing Joy choice for the timing of interventions including mobilization to a chair. Timing of mobilization might require considering the timing of other interventions and, therefore, requires a coordinated approach from the team. The team also suggested the provision of communication tools to encourage Joy to have more agency in her care, communicate her needs, choices, and questions about this proposed intervention and others.

### Social exchange and reciprocity

The team discussed the need for positive encounters to grossly outweigh negative ones—for example, for every negative interaction, there needs to be several counteracting positive interactions. The healthcare team might, therefore, benefit from a team meeting to manage expectations and consistency with Joy and Agnes. The team may benefit from some coaching about how to navigate difficult conversations with patients and families.

### Empathy, caring and acceptance

The team discussed the importance of involving Joy and Agnes in collaborative decision making and integrating Joy’s goals in the care plan. They recommend asking questions, using active listening, and demonstrating caring through acceptance of her situation as much as she shares this with them. The team emphasized the importance of a consistent approach for collaborative and patient centred goal setting with all care team members.

### Genuineness and openness

The team discussed being transparent and open during goal setting with Joy and Agnes, and to acknowledge genuine sharing of feelings and experiences when shared.

### Reading and responding to emotions

The team discussed the importance of active listening practice for clinicians to help develop skills needed to respond therapeutically to emotionally charged situations. Clinicians might acknowledge the initial emotional expression (i.e., anger), then work with Joy and Agnes to brainstorm acceptable solutions to the problem (i.e., collaboratively plan an acceptable time to try sitting, ensuring Joy and Agnes understand the implications of missing the therapy, and that the staff understand the barriers to performing the therapy), and then carry out the planned response.

### Avoiding coercion

In this case scenario, Joy declined mobilization therapy, and Agnes expressed frustration over the team not mobilizing her daughter. The team discussed that instead of being reactive to the negative emotions expressed in the moment, after acknowledging the emotional expression, the healthcare providers can also note the incongruence in expectations and goals expressed signalling a lack of collaboration in the goals. The team can then express willingness to work collaboratively with Joy and Agnes towards a mutually acceptable plan. This might include involvement of other professionals such as bioethicists, spiritual care, or psychiatry as appropriate. This might also include sharing with Joy and Agnes institutional limitations or resources so they can work towards a feasible plan.

### Setting limits and interpreting boundaries

The team discussed that inconsistencies in approach among the care team can lead to further anger and frustration. They also suggested that mutually derived care plans be posted in Joy’s room for her, her family, and the healthcare team to see and update on a regular basis. It might also be helpful to have institutional policies and posters supporting a violence-free workplace in visible areas on the unit.

## Discussion

Using an ICU-based fictional exemplar and crisis intervention principles, this paper describes the application of de-escalation principles of a crisis intervention program for healthcare. Beyond individual level interventions to manage difficult situations in the ICU, this exercise highlighted several key implications for healthcare leaders in critical care. These include the importance of crisis intervention training that transcends conflict management, the importance of team training, and support of organization policies that include patients and family as partners.

Acknowledging difficult emotions such as anger and frustration was a key strategy discussed and can communicate empathy, caring, and responding to emotions expressed by patient and family. Patient and family conflict with healthcare staff is not uncommon in ICU.^12^ Crisis management rooted in trauma-informed care principles can help clinicians move beyond episodic conflict management and towards relationship recovery and the provision of humanistic care. Humanistic care, which includes expressing care, concern, attention to individual needs, and respecting patient rights has the potential to enhance patients’ ability to deal with stress and promote recovery.^13,14^

In our example, crisis intervention de-escalation strategies at the individual level were discussed including active empathic listening, provision of choice, and collaborative goal setting. These interventions, though important for individual clinicians to practice, are likely to be less effective if not practiced consistently as a team and endorsed by health leaders. The absence of a team approach in healthcare can place patients at risk for fractures in care and safety.^15^ A recent systematic review of interventions to improve team effectiveness in healthcare identified three types of interventions to improve team functioning including general team training as well as tools that stimulate team processes.^16^ As in our fictional exemplar, the authors of this review found that most studies evaluated interventions focussed on non-technical skills and found improvements in team functioning.^16^ Furthermore, inconsistencies between team members can aggravate frustration and resentment between healthcare team members, patients, and families. Team crisis intervention training as part of a robust workplace violence prevention program, and as part of the creation of a trauma-informed ICU, can protect patients, catapult individual clinician skill development, and improve ICU team cohesion and functioning.

In this exercise, we identified the need for the entire team to have relational skills training, again to have a consistent approach with patients and families. This training can be tailored to the local organization based on specific needs and availability of resources. The Canadian Alliance of Nurse Educators Using Simulation (CAN-Sim) web site houses a variety of education simulations including a ventilated patient scenario.^17^ In addition to in person training, virtual simulation can be considered as it has been found to be an effective modality for learning new clinical skills that can be uncomfortable to practice as a novice at the bedside.^18^

Working through the exemplar highlighted policy level implications of de-escalation and relational training using a crisis intervention framework. These included processes of care that improve team cohesion and the proactive and consistent inclusion of patients and families in decision making. Supporting staff training in skills that support patient and family-centred communication skills is consistent with the LEADS framework.^19^ As a guiding framework for healthcare leaders, LEADS addresses personal, interpersonal, operational, and strategic leadership capabilities and context that among other priorities shifts models of care from provider-centric, to one that is patient-centred.^20^

As in the fictional exemplar, the use of mechanical ventilation in ICU renders patients voiceless. Communication difficulty has been reported to be one of the most frustrating and dehumanizing experiences reported by ICU survivors.^21,22^ Despite their right to communicate,^23^ barriers to the provision of communications support in the ICU include a lack of tools and training.^24^ Concurrent training in communication strategies for non-vocal patients as well as institutional access to communication tools for ICU patients can help ensure that patient rights are maintained and that they are included in conversations about their care. Finally, teamwork and patient/family engagement are known contributors to quality care in ICU,^25^ and particularly for patients with prolonged admission, such as the patient in our exemplar case.^26^ Organizational and unit-based collaborative team processes that create increased team cohesion such as multiprofessional rounds and documentation platforms should be created to keep the team, patient, and family on the same page.

## Conclusion

Using a fictional exemplar, this paper described one way to improve ICU clinician relational management skills including de-escalation towards the creation of a trauma-informed ICU. Key messages include a call to action for leadership support of training for all members of critical care teams to enhance skilled relational engagement of patients and families in the ICU to improve care and prevent (re-) traumatization. Key messages also included the importance of providing crisis intervention training that transcends conflict management and leadership support of organization policies that include patients and families as partners in their care.

Box 1. Summary of recommendations to support patient engagement and the creation of a trauma-informed ICU

• Endorse crisis intervention training that includes relational skill building for all staff.
• Support processes of care that formalize collaboration between multiprofessional team members including rounding and documentation practices.
• Support processes of care that formalize patient and family inclusion in decision making, including the provision of tools and training for all staff to support patient communication.
